# Long-term clinical clerkship improves medical students' attitudes toward team collaboration

**DOI:** 10.5116/ijme.633f.e97a

**Published:** 2022-10-31

**Authors:** Kazunori Ganjitsuda, Masami Tagawa, Kazuya Tomihara, Takuya Saiki, Makoto Kikukawa, Akiteru Takamura, Hitoaki Okazaki, Yasushi Matsuyama, Rika Moriya, Hiroki Chiba, Yasushi Takagi, Hitoshi Setoyama, Akihiro Tokushige, Hidetaka Yokoh

**Affiliations:** 1 Center for Innovation in Medical and Dental Education, Graduate School of Medical and Dental Sciences, Kagoshima University, Japan; 2 Department of Psychology, Faculty of Law, Economics, and Humanities, Kagoshima University, Japan; 3 Medical Education Development Center, Gifu University, Japan; 4 Department of Medical Education, Kyushu University, Japan; 5 Department of Medical Education, University of Toyama, Japan; 6 Medical Education Center, Jichi Medical University, Japan; 7 Department of Medical Education, Research and Development Center for Medical Education, Kitasato University, Japan; 8 Education Office, Showa University, Japan; 9 Kagoshima Prefectural Comprehensive Health Center, Japan

**Keywords:** Medical student, teamworking, interprofessional education, transprofessional education, clinical clerkship

## Abstract

**Objectives:**

To examine the related factors associated with medical students' attitudes toward team collaboration.

**Methods:**

This cross-sectional study targeted medical students, residents, and doctors. A survey was conducted from 2016 to 2017 using the Japanese version of the Jefferson Scale of Attitudes Toward Interprofessional Collaboration (JeffSATIC-J), which evaluated "working relationship" and "accountability." We analyzed 2409 questionnaire responses with JeffSATIC-J items and the gender item. Analysis of variance was used for factors associated with the JeffSATIC-J score and Spearman's rank correlation coefficient for the relationship between educational intervention and the JeffSATIC-J score.

**Results:**

First-year students’ scores were the highest (F_(2, 2045)_ = 13.42 to 18.87, p < .001), and female students’ scores were significantly higher than those of male students (F_(1, 2045)_ = 21.16 to 31.10, p < .001). For residents' scores, the institution was not a significant variable. Female "accountability" scores were significantly higher than those of males (F _(1,108)_ = 4.95, p = .03). Gender was not a significant variable for doctors' scores. Sixth-year students' scores were significantly correlated with the length of clinical clerkship (r_(5)_=.78 to .96, p<.05), with the exception of females' "working relationship" scores. The medical school with the highest JeffSATIC-J scores had the longest clinical clerkship in the community.

**Conclusions:**

These results indicate that long-term clinical clerkship in the community at higher grades is important in improving medical students' attitudes toward team collaboration. A qualitative study is required to confirm our findings.

## Introduction

Collaborative work between multiple health professions provides high-quality, comprehensive health services and results in optimum outcomes with low patient mortality and high patient satisfaction.^1^^-^^4^ However, collaborations are often hindered by undesirable expectations of other professionals' contributions and lack of communication. These challenges might arise from differences in recognition of one's own role and the roles of other health professionals on the team, insufficient trust, lack of mutual respect for other professionals, and misunderstandings about collaborative work.^2^^,^^5^^-^^8^

To raise awareness of team collaboration and respect for other professionals, various educational strategies have been introduced in undergraduate medical education.^9^^-^^11^ Interprofessional education (IPE), defined by the Center for Advancement of Interprofessional Education (CAIPE) as "occasions when two or more professions learn with, from and about each other to improve collaboration and quality of care," ^12^ is considered an effective strategy for improving attitudes toward and readiness for interprofessional work.^9^^-^^11^ In addition to IPE in the classroom, education with multiple professionals in the context of clinical practice, referred to as transprofessional education, is an advanced, effective strategy for team collaboration.^13^

To evaluate IPE outcomes, a variety of scales have been developed and used for medical and other health professional students.^14^ The Collaborative Healthcare Interdisciplinary Relationship Planning Scale has been validated to evaluate interdependence, recognition, empathy, sharing, dominance, organizational climate, and respect,^15^ and the Readiness for Interprofessional Learning Scale to evaluate teamwork and collaboration, professional identity, and roles and responsibilities.^16^^,^^17^ Previous studies using these scales revealed that students' attitudes and readiness for multiprofessional shared learning were improved after IPE courses.^18^^-^^20^

Meanwhile, Hojat focused on attitudes toward team collaboration among health professionals and developed three scales applicable to students and practicing professionals: the Jefferson Scale of Attitudes toward Physician-Nurse Collaboration (JSAPNC),^21^^-^^23^ the Scale of Attitudes Toward Physician-Pharmacist Collaboration (SATP^2^C),^24^ and the Jefferson Scale of Attitudes Toward Interprofessional Collaboration (JeffSATIC).^25^ Unlike the JSAPNC and SATP^2^C, JeffSATIC can be used for all health professions. Validation of JeffSATIC indicated that it evaluates two factors.^25^ The first factor, named "working relationship," consists of items such as "All health professionals have their own special expertise to render quality care to their patients/clients" and "Health professionals should be made aware that their colleagues in other health-related disciplines can contribute to the quality of care," and evaluates the understanding of what constitutes outstanding collaborative work. The second factor, named "accountability," consists of items such as "Health professionals should not question decisions made by colleagues even if they feel that it might have detrimental effects on the patient/client (reverse count)," and evaluates one's attitude toward the accomplishment of collaboration with others. Cronbach's α coefficients of the JeffSATIC ranged from 0.84 to 0.90 in three studies.^25^

Previous research with Jefferson scales revealed that physicians had more negative attitudes toward collaboration than nurses^26^^,^^27^ and pharmacists.^24^^,^^28^ In addition, region was identified as an influential factor as health professionals in America and Israel had more positive collaborative attitudes than those in Italy and Mexico.^26^^,^^27^ Furthermore, female medical and other health professional students had higher interprofessional collaborative attitudes than male students.^25^^,^^29^

Meanwhile, Japan, which is characterized by doctors' high status in team collaboration, has no reports using JeffSATIC. Therefore, we investigated factors related to medical students' and doctors' perceptions of team collaboration using JeffSATIC.

The study had three objectives. The first was to establish the Japanese version of the JeffSATIC (JeffSATIC-J), enabling evaluation of attitudes toward interprofessional team collaboration among health professionals and trainees. The second was to reveal young medical trainees' collaborative attitudes with the JeffSATIC-J by evaluating students at different phases of the undergraduate program and residents at the end of the program. The third was to elucidate factors related to the JeffSATIC-J scores.

## Methods

### Study design and participants

This was a multi-institutional cross-sectional study targeting medical students immediately after their admission (first year), those about to start their clinical clerkship courses (fourth year), or those who had finished all clinical clerkship courses (sixth year), as well as residents who had finished a 2-year residency program and medical doctors.

Medical students in Japan have to pass the Common Achievement Test prior to their first clinical clerkship course to obtain permission to perform medical procedures on patients as members of the medical care team. All fourth-year students in this study were in their preclinical years, and their learning was limited to observation of clinical practice, and simulations, besides classroom lectures and group discussions.

This research was approved by the Ethics Committee of Kagoshima University Graduate School of Medical and Dental Sciences, as well as by the ethics committees of all other participating institutions. This investigation was conducted according to the principles expressed in the Declaration of Helsinki. Participants were informed that their cooperation was voluntary, that no personal assessment would be conducted, that no reward would be provided, that their identity and data would be protected and that the results may be published. Returning a completed questionnaire was regarded as providing consent to participate in the study.

### Measures

The original English version of the JeffSATIC comprises 20 items, and each item is scored on a 7-point Likert scale ranging from 1 (strongly disagree) to 7 (strongly agree).^25^ It was translated into Japanese with the permission of the original JeffSATIC authors. Next, a translation expert back-translated the JeffSATIC-J into English, and the JeffSATIC authors confirmed its equivalence with the original items.

Cronbach's α coefficient was obtained, and exploratory factor analysis was conducted to examine the reliability and subscale structure of the JeffSATIC-J.

### Data collection method

An anonymous written questionnaire survey containing the JeffSATIC-J and items inquiring about age and gender, and for doctors, length of clinical experience, was conducted from 2016 to 2017. Authors from each medical school explained the purpose and ethical considerations of this research and collected the completed questionnaires.

In consideration of the possible effects on students' education, fourth- and sixth-year medical students were asked to complete the questionnaire at the end of the academic year or after all teamwork courses were finished.

In addition to the questionnaire, information on courses related to teamwork and clinical practice that fourth- or sixth-year students attended in each medical school was also collected from course directors and by checking the syllabi.

### Study setting

There are 82 medical schools in Japan; all have a 6-year program required by University Establishment Standards and are certified by university accreditation bodies. All medical schools accept high school graduates who are 18 years or older, implementing a variety of admission policies and selection methods. As regionality and organizational characteristics are known as influential factors for team collaboration,30 seven medical schools were selected for this research based on their region, founders, and school mission. Three of them were national schools (Gifu University School of Medicine, GU; Kyushu University School of Medicine, KyuU; Kagoshima University School of Medicine, KaU), and four were private schools (Showa University School of Medicine, SU; Kitasato University School of Medicine, KiU; Kanazawa Medical University School of Medicine, KMU; Jichi Medical University School of Medicine, JMU). Residency programs in four medical school hospitals (KyuU, KaU, KiU, KMU) were also selected. In addition, doctors from one medical school hospital (KaU) were selected.

JMU is a unique medical school that admits students from across the country, and students receive scholarships from their home prefectures that completely cover their entrance fees and tuition. After graduation, students are obligated to work for 9 years in their home prefectures, which includes 4 or 5 years at hospitals or clinics in rural areas.

Responses were collected from 2514 of the 3017 target participants. The number of responses with all JeffSATIC-J items answered was 2426 (80.4%), and the number with all JeffSATIC-J items and the gender item answered was 2333 (77.3%).

The number of responses with 16 or more JeffSATIC-J items answered was 2504 (83.0%), and the number with 16 or more JeffSATIC-J items and the gender item answered was 2409 (79.8%). Among them, the number of medical student responses from GU, KyuU, KaU, SU, KiU, KMU, and JMU were 299 (97.4%), 266 (80.4%), 291 (81.7%), 356 (95.4%), 309 (90.6%), 242 (71.2%), and 324 (92.6%), respectively. The number of responses from first-, fourth-, and sixth-year medical students, residents, and doctors were 755 (95.1%), 647 (78.8%), 685 (87.5%), 116 (60.4%), and 206 (48.2%), respectively (Table 1).

The female/male ratios among first-, fourth-, and sixth-year students, residents, and doctors were 248/507 (0.49), 209/438 (0.48), 224/461 (0.49), 41/75 (0.55), and 49/157 (0.31), respectively. Doctors' average years of clinical experience were 13.95 (SD: 7.96, Range: 1-36).

### Data analysis

According to CAIPE^12^ and Harden's educational steps^13^ teamworking-related courses were classified in three ways. The first is education for medical students only (uniprofessional), in which medical students learn without any other health professional students (UPE). The second is multiprofessional education without mutual interaction between students of different professions (MPE) (e.g., classroom lectures). The third is multiprofessional education with mutual interaction between students of different professions, in which students learn from and about each other (IPE) (e.g., case-based small group discussions, role-playing).

In addition, courses in hospitals or other healthcare institutions were classified in two ways. The first is clinical observation, in which students observe interprofessional health care work but do not have any role in the delivery of care, and the second is clinical clerkship, in which students have opportunities to function as members of the health care team in the delivery of care.

Returned questionnaire responses with 16 or more JeffSATIC-J items and the gender item answered were used for the following analyses. Following the JeffSATIC scoring algorithm, unanswered items were replaced with the mean score of other items completed by the same respondent.

After the validation of the JeffSATIC-J, the total score and subscale scores were analyzed by the institute, learning year group, and gender. To examine the factors associated with the JeffSATIC-J score, an analysis of variance (ANOVA) followed by the Bonferroni procedure was conducted. In addition, an unpaired two-sample Student's t-test was used to determine the gender differences in the medical doctors' sample.

To examine the relationship between educational intervention and the JeffSATIC-J score, Spearman's rank correlation coefficient for the length of teamwork or clinical courses was analyzed. All statistical analyses were done at a significance level of 0.05 using SPSS version 21.

## Results

### Validity and reliability of JeffSATIC-J

To identify the underlying dimensions, we conducted an exploratory factor analysis of the 20 items of JeffSATIC-J using 2426 complete responses. The exploratory factor analysis identified two factors with eigenvalues greater than 1.0. The complete response cases indicated a first factor (eigenvalue=6.50, accounting for 32.5% of total variances) and second factor (eigenvalue=2.40, accounting for 12.0% of total variances) (Table 2).

**Table 1:** Number of respondents and average age of groups in each institution

Institution		First-year medical students					Fourth-year medical students					Sixth-year medical students					Second-year residents					Medical doctors			
		n	Response rate (%)	Age (years)			n	Response rate (%)	Age (years)			n	Response rate (%)	Age (years)			n	Response rate (%)	Age (years)			n	Response rate (%)	Age (years)	
				Mean	SD				Mean	SD				Mean	SD				Mean	SD				Mean	SD
GU	Total	107	98.2	19.1	2.5		104	100	24.0	5.4		88	93.6	27.0	9.0										
	Female	38		18.6	1.5		28		21.8	1.0		20		23.9	0.4										
	Male	69		19.3	2.8		76		24.8	6.1		68		27.9	10.0										
KyuU	Total	104	94.5	19.5	2.8		75	63.0	22.8	1.7		87	85.3	24.6	1.8		26	43.3	29.1	4.4					
	Female	22		19.8	3.6		12		22.8	1.4		14		24.1	1.0		10		29.1	5.8					
	Male	82		19.4	2.6		63		22.8	1.7		73		24.7	1.9		16		29.1	3.1					
KaU	Total	95	90.5	19.2	1.3		85	68.5	23.6	3.2		111	87.4	25.1	2.3		23	79.3	28.4	2.9		206	48.2	39.7	8.2
	Female	33		19.1	1.0		38		22.7	1.1		45		24.7	2.1		5		28.0	3.0		49		35.2	6.0
	Male	62		19.2	1.5		47		24.3	4.0		66		25.4	2.4		18		28.5	2.9		157		41.1	8.3
SU	Total	119	100	18.8	0.8		109	87.9	23.1	3.0		128	98.5	25.0	1.4										
	Female	37		18.5	0.6		30		23.4	5.5		33		24.6	0.9										
	Male	82		18.9	0.8		79		22.9	1.2		95		25.1	1.5										
KiU	Total	120	99.2	19.4	1.9		86	78.2	23.9	3.3		103	93.6	24.6	1.8		36	63.2	27.1	1.7					
	Female	53		18.9	1.4		37		23.4	2.7		49		24.2	1.7		14		26.8	0.7					
	Male	67		19.7	2.2		49		24.3	3.7		54		24.9	1.8		22		27.4	2.1					
KMU	Total	94	87.9	19.9	1.4		72	61.0	24.5	2.7		76	66.1	26.6	4.2		31	67.4	28.5	2.5					
	Female	37		19.5	1.3		34		23.9	2.4		36		26.5	5.4		12		27.6	1.6					
	Male	57		20.2	1.4		38		24.9	3.0		40		26.7	2.7		19		29.0	2.8					
JMU	Total	116	94.3	19.1	1.3		116	95.1	22.3	1.5		92	87.6	24.4	1.2										
	Female	28		19.1	1.3		30		22.1	1.0		27		24.2	0.9										
	Male	88		19.1	1.2		86		22.4	1.6		65		24.4	1.3										
Overall	Total	755	95.1	19.3	1.9		647	78.8	23.4	3.3		685	87.5	25.2	3.9		116	60.4	28.2	3.0		206	48.2	39.7	8.2
	Female	248		19.0	1.6		209		22.9	2.8		224		24.7	2.6		41		27.7	3.3		49		35.2	6.0
	Male	507		19.4	2.0		438		23.6	3.6		461		25.5	4.3		75		28.4	2.8		157		41.1	8.3

GU: Gifu University School of Medicine; KyuU: Kyushu University School of Medicine; KaU: Kagoshima University School of Medicine; SU: Showa University School of Medicine; KiU: Kitasato University School of Medicine; KMU: Kanazawa Medical University School of Medicine; JMU: Jichi Medical University School of Medicine; SD: standard deviation.

**Table 2:** Factor loadings for promax rotated two-factor solutions for 20 JeffSATIC-J items

Items		Working relationship	Accountability
		n= 2426	
18	All health professionals have their own special expertise to render quality care to their patients/clients.	.730*	-.063
11	All health professionals should contribute to decisions regarding improving care of their patients/clients.	.723*	.107
13	Health professionals should be made aware that their colleagues in other health-related disciplines can contribute to the quality of care.	.716*	.063
7	Collaborative practice always works best when health professionals develop working relationships to achieve agreed upon goals.	.683*	-.217
14	Health professionals should be involved in making policy decisions concerning their work.	.676*	.097
6	All health professionals can contribute to decisions regarding the well-being of patients/clients.	.655*	-.032
10	Interprofessional collaboration which includes mutual respect and communication improves the work environment.	.623*	.237
2	All health professionals should have responsibility for monitoring the effects of interventions on their patients/clients.	.618*	-.149
20	During their education, all health profession students should experience working in teams with other health profession students in order to understand their respective roles.	.590*	.086
17	Medical errors will be minimized when collaboration exists among health professionals.	.561*	-.134
1	Health professionals should be viewed as collaborators rather than superiors or subordinates.	.538*	-.023
4	Academic institutions should develop interdisciplinary educational programs to enhance collaborative practice.	.537*	-.007
5	Health professionals should not question decisions made by colleagues even if they feel that it might have detrimental effects on the patient/client.	-.013	.789*
9	The primary function of other health professionals is to follow, without question, orders by the physician who are treating the patients/clients.	.022	.784*
16	To promote the best interest of the patient/client, health professionals should use their own judgment rather than consulting their colleagues in other health-related disciplines.	.028	.730*
8	Interdisciplinary education and interprofessional collaboration are not linked to one another.	.005	.722*
15	Because of role differentiation, there are not many overlapping areas of responsibility among health professionals in providing care to their patients/clients.	-.077	.716*
12	Job satisfaction has nothing to do with interprofessional collaborative practices.	.094	.651*
3	Teamwork in healthcare cannot be an outcome of interdisciplinary education.	-.167	.638*
19	Health professionals working together cannot be equally accountable for the care/ service they provide.	-.117	.482*
Eigenvalue		6.50	2.40
% Variance		32.5%	12.0%

Items are listed by the order of magnitude of the factor coefficients within each factor. *Factor coefficients greater than .40. Jefferson is the sole copyright holder of the JeffSATIC. Permission to use the instrument and its translated versions must be requested from Jefferson, or the creator of the instrument: mohammadreza.hojat@jefferson.edu

Additionally, the factor structures of these items were consistent with those reported by Hojat and colleagues.^25^ Therefore, in accordance with their study, we interpreted the factors to represent the "working relationship" and "accountability," respectively.

The α coefficient for the JeffSATIC-J total, "working relationship," and "accountability" subscale scores were 0.879, 0.862, and 0.832, respectively. We confirmed the reliability of the JeffSATIC-J and determined it was equivalent to the original scale.

### JeffSATIC-J scores and related factors

Average JeffSATIC-J total scores of male and female first-, fourth-, and sixth-year students, residents, and doctors, the average "working relationship" subscale score, and the "accountability" subscale score are shown in Tables 3 and 4.

The average JeffSATIC-J total score for medical students was 107.4 (SD 13.9). A three-way ANOVA indicated that the main effects of gender were significant on the variables of students’ total (F _(1, 2045)_ = 31.10, p < .001) as well as factor scores (“working relationship”: F _(1, 2045)_ = 21.16, p < .001; “accountability”; F _(1, 2045)_ = 21.56, p < .001), as indicated by the higher scores of female students than those of male (Table 3).

The main effects of learning year were also significant on these variables (“total”: F _(2, 2045)_ = 18.87, p < .001; “working relationship”: F _(2, 2045)_ = 13.42, p < .001, “accountability”: F _(2, 2045)_ = 13.91, p < .001, Table 3). A post hoc Bonferroni procedure of the total and two-factor subscale scores indicated that first-year students' scores were significantly higher than those of other learning year groups (p < .001), whereas no significant difference was revealed between fourth- and sixth-year students.

Additionally, the main effects of institution (“total”: F _(6, 2045)_ = 8.66, p < .001; “working relationship”: F _(6, 2045)_ = 4.36, p < .001, “accountability”: F _(6, 2045)_ = 10.37, p < .001), and the interaction between institution and learning year (“total”: F _(12, 2045)_ = 9.04, p < .001; “working relationship”: F _(12, 2045)_ = 7.18, p < .001; “accountability”: F _(12, 2045)_ = 6.10, p < .001) were significant (Table 3). Concerning differences among institutions in each learning year, it is difficult to find a clear tendency (Table 4). However, several significant differences were observed in first-year students' JeffSATIC-J total and two-factor subscale scores among the seven medical schools (p < .05), even though the students completed the questionnaire shortly after admission and prior to taking any teamwork courses. KyuU had the highest fourth-year student scores, whereas JMU had the highest sixth-year student scores. A two-way ANOVA of residents' scores indicated that institution was not a significant variable. Female “accountability” scores were significantly higher than male scores (F _(1,108)_ = 4.95, p = .03) (Table 3). An unpaired two-sample Student's t-test of medical doctors' total scores indicated that there was no significant difference between males and females (t _(204)_ = .38 p = .70) (Table 3). The two subscale scores were also not significant.

### Educational characteristics of medical schools

As shown in Table 5a, all medical schools implemented teamwork courses, but educational strategies, academic year offered (e.g., first-year course, third-year course), length, and the sequence of the courses varied among institutions. KyuU offered teamwork courses as electives, whereas other schools offered them as required courses. The length of required teamwork courses in 6 years was from 6 (GU) to 37.5 (KaU) hours.

All medical schools implemented IPE. Five medical schools (GU, KyuU, KaU, SU, KMU) implemented IPE courses only in the preclinical years, KiU implemented an MPE course in the preclinical years and an IPE course in the clinical years, and JMU implemented a 5.8-hour IPE course after all clinical clerkship courses were completed. SU implemented IPE courses in the first, third, and fourth years, with the longest learning hours.

The length of clinical clerkship courses varied from 45 to 74 weeks, with JMU having the longest course duration. During clinical clerkship courses, students experienced teamwork with multiple professionals (transprofessional care) by playing the role of a team member in the context of real practice, which is not permitted in preclinical years in Japanese medical schools. All JMU students train in community medicine for 7 weeks in a rural area, where students go to work after graduation.

### Fourth-year students' JeffSATIC-J scores and relationship with the length of courses in preclinical years

Medical schools were ranked in order of the length of their teamwork or clinical observation courses. As KyuU provides elective courses of varying lengths, the same ranking was given to medical schools if the course length was the same or within the range of KyuU courses Table 5a 

Spearman's rank correlation between fourth-year students' JeffSATIC-J scores and length of courses was analyzed. As shown in Table 5a fourth-year female students' "working relationship" (r _(5)_ = .67, p < .05) and "accountability" (r _(5)_ = .67, p < .05) scores were significantly correlated with the length of the IPE course provided in the fourth year, but not with any other courses or courses in previous years. Fourth-year male students' "accountability" scores were not correlated with any courses in preclinical years.

### Sixth-year students' JeffSATIC-J scores and relationship with the length of courses in preclinical and clinical years

JMU implemented the longest clinical clerkship courses including training in community hospitals and clinics, and IPE immediately after the clinical clerkship course, but did not offer these in preclinical years. The length of clinical clerkship courses was significantly correlated with sixth-year students’ “accountability” scores (r _(5)_ = .88, p < .01) and male students’ “working relationship” scores (r _(5)_ = .83, p < .05) Table 5b. Teamwork courses in preclinical years were negatively correlated with sixth-year students' scores, from r _(5)_ = -.87 to -.76 (p < .05).

**Table 3:** Group comparison of JeffSATIC-J scores and related factors

Status	Groups	n	Total			Working relationship			Accountability		
			Mean	SD	Statistical significance	Mean	SD	Statistical significance	Mean	SD	Statistical significance
Medical students	Learning year										
	First-year	755	110.3 a	11.7	< .001	67.7 a	7.9	< .001	42.6 a	5.9	< .001
	Fourth-year	647	105.9 b	14.6	F_(__2, 2045)_ = 18.87	64.9 b	9.5	F_(__2, 2045)_ = 13.42	41.0 b	7.6	F_(__2, 2045)_ = 13.91
	Sixth-year	685	105.6 b	14.9		65.4 b	9.9		40.2 b	8.3	
											
	Institution										
	GU	299	108.8 a	13.0	< .001	66.7 a, b	8.5	< .001	42.1 a, b	6.3	< .001
	KyuU	266	108.5 a. b	12.0	F_(__6,2045)_ = 8.66	66.7 a, b	8.0	F_(__6,2045)_ = 4.36	41.8 a, b	6.0	F_(__6,2045)_ = 10.37
	KaU	291	108.3 a, b	13.1		66.5 a, b	8.6		41.8 a, b	6.8	
	SU	356	105.5 b, c	15.8		65.9 a, b	10.6		39.5 c	9.3	
	KiU	309	105.5 b, c	13.7		64.7 b	9.0		40.8 b, c	6.6	
	KMU	242	104.4 c	15.8		64.7 b	10.2		39.7 c	8.7	
	JMU	324	110.4 a	12.6		67.0 a	8.4		43.4 a	6.2	
											
	Gender										
	Female	681	109.5 a	13.1	< .001	67.2 a	8.5	< 001	42.3 a	6.9	< .001
	Male	1406	106.3 b	14.2	F_(__1,2045)_ = 31.10	65.5 b	9.4	F_(__1,2045)_ = 21.16	40.8 b	7.6	F_(__1,2045)_ = 21.56
											
Residents	Institution										
	KyuU	26	104.3	12.3	n.s.	62.3	8.9	n.s.	42.0	5.2	n.s.
	KaU	23	104.7	18.9	F_(__3,108)_ = 1.19	63.1	13.3	F_(__3,108)_ = 0.41	41.7	6.8	F_(__3,108)_ = 2.25
	KiU	36	96.4	15.7		59.8	10.7		36.6	9.1	
	KMU	31	102.2	13.9		63.0	9.8		39.2	9.5	
											
	Gender										
	Female	41	103.4	16.1	n.s.	61.5	11.7	n.s.	41.9 a	5.9	p = 0.03
	Male	75	100.3	15.5	F_(__1,108)_ = 0.54	62.1	10.1	F_(__1,108)_ = 0.31	38.2 b	9.1	F_(__1,108)_ = 4.95
											
Medical doctors	Gender										
	Female	49	107.9	12.5	n.s.	65.1	8.7	n.s.	42.8	5.5	n.s.
	Male	157	107.0	15.0	t _(204)_ = 0.38	64.7	9.6	t _(204)_ = 0.27	42.3	6.7	t _(204)_ = 0.46

p for comparison of two, three or more groups using the t-test and ANOVA, respectively. Means denoted by a different letter indicate significant differences between groups.

JeffSATIC-J: Japanese version of the Jefferson Scale of Attitudes Toward Interprofessional Collaboration; GU: Gifu University School of Medicine; KyuU: Kyushu University School of Medicine; KaU: Kagoshima University School of Medicine; SU: Showa University School of Medicine; KiU: Kitasato University School of Medicine; KMU: Kanazawa Medical University School of Medicine; JMU: Jichi Medical University School of Medicine; SD: standard deviation.

**Table 4:** JeffSATIC-J score by group in each institution

Institution	First-year medical students			Fourth-year medical students			Sixth-year medical students			Second-year residents			Medical doctors		
	n	Mean	SD	n	Mean	SD	n	Mean	SD	n	Mean	SD	n	Mean	SD
Total															
GU	107	113.1 a, b	9.9	104	105.8 b, c	13.5	88	107.3a, b	14.2						
KyuU	104	106.5 b, c	10.7	75	113.5 a	11.4	87	106.6 a, b	12.6	26	104.3	12.3			
KaU	95	109.5 a, b	11.4	85	108.8 a, b	13.9	111	106.9 b	13.6	23	104.7	18.9	206	107.2	14.4
SU	119	115.0 a	9.0	109	99.9 d	15.6	128	101.4 b	16.7						
KiU	120	111.2 a, b	11.9	86	101.6 c, d	14.2	103	102.1 b	12.9	36	96.4	15.7			
KMU	94	103.7 c	14.0	72	108.3 a, b	16.2	76	101.6 b	16.7	31	102.2	13.9			
JMU	116	111.2 a, b	11.2	116	106.2 b, c	13.0	92	114.6 a	12.3						
Overall	755	110.3	11.7	647	105.9	14.6	685	105.6	14.9	116	101.4	15.7	206	107.2	14.4
Working relationship															
GU	107	69.7 a, b	7.0	104	64.6 a, b	8.3	88	65.6 a, b	9.3						
KyuU	104	65.8 b, c	7.7	75	68.7 a	7.8	87	66.1 a, b	8.3	26	62.3	8.7			
KaU	95	67.3 a, b	7.7	85	66.3 a	8.6	111	65.9 a, b	9.3	23	63.1	13.0	206	64.8	9.4
SU	119	70.8 a	6.0	109	62.3 b	10.9	128	64.6 a, b	12.0						
KiU	120	68.0 a, b	8.3	86	62.0 b	9.0	103	63.2 b	8.6	36	59.8	10.6			
KMU	94	63.4 c	8.9	72	67.9 a	10.1	76	63.2 b	11.2	31	63.0	9.6			
JMU	116	67.6 a, b, c	7.6	116	64.2 a, b	9.0	92	69.6 a	7.6						
Overall	775	67.6	8.0	647	64.9	9.5	685	65.4	9.9	116	61.9	10.6	206	64.8	9.4
Accountability															
GU	107	43.3 a	4.8	104	41.2 a, b	6.9	88	41.7 a, b	7.0						
KyuU	104	40.7 a, b	5.7	75	44.8 a	5.2	87	40.5 b, c	6.2	26	42.0	5.1			
KaU	95	42.1 a, b	7.2	85	42.6 a, b	6.2	111	41.0 b	6.8	23	41.7	6.6	206	42.4	6.4
SU	119	44.2 a	5.0	109	37.6 c	8.6	128	36.8 c	11.1						
KiU	120	43.2 a, b	5.1	86	39.6 b, c	7.0	103	38.9 b, c	7.0	36	36.6	9.0			
KMU	94	40.3 b	7.1	72	40.4 b, c	10.5	76	38.4 b, c	8.6	31	39.2	9.3			
JMU	116	43.6 a, b	5.7	116	42.0 a, b	6.1	92	45.0 a	6.6						
Overall	755	42.6	5.9	647	41.0	7.6	685	40.2	8.3	116	39.5	8.2	206	42.4	6.4

Means denoted by a different letter indicate significant differences between institutions. JeffSATIC-J: Japanese version of the Jefferson Scale of Attitudes Toward Interprofessional Collaboration; GU: Gifu University School of Medicine; KyuU: Kyushu University School of Medicine; KaU: Kagoshima University School of Medicine; SU: Showa University School of Medicine; KiU: Kitasato University School of Medicine; KMU: Kanazawa Medical University School of Medicine; JMU: Jichi Medical University School of Medicine; SD: standard deviation.

**Table 5a:** Spearman's rank correlation coefficient (r) between JeffSATIC-J score and length of courses related to teamworking or clinical practice (A) JeffSATIC-J score of fourth-year students

Variables		Institution	Courses related to teamwork														Clinical practice
			First year			Second year	Third year		Fourth year			First-fourth year					First-fourth year
			UPE	MPE	IPE	UPE	UPE	IPE	UPE	MPE	IPE	UPE	MPE	IPE	M/IPE	Total	Observation
			(hrs)	(hrs)	(hrs)	(hrs)	(hrs)	(hrs)	(hrs)	(hrs)	(hrs)	(hrs)	(hrs)	(hrs)	(hrs)	(hrs)	(hrs)
Length of courses		GU	0	3	0	0	0	0	0	1.25	1.75	0	4.25	1.75	6	6	0
		KyuU	0	0	0	0	0	0	0	9‐22	7‐23	0	9‐22	7‐23	16-45	16-45	0
		KaU	0	0	0	15	0	0	0	0	22.5	15	0	22.5	22.5	37.5	37
		SU	0	0	19.5	0	0	4.5	0	0	4.5	0	0	28.5	28.5	28.5	0
		KiU	0	22.5	0	0	0	0	0	0	0	0	22.5	0	22.5	22.5	91
		KMU	1.5	0	0	0	0	0	1.5	0	20	3	0	20	20	23	200
		JMU	0	0	0	0	1.2	0	0	0	0	1.2	0	0	0	1.2	0
Overall	Total	r	.20	-.49	-.61	.41	.00	-.61	.20	.40	.67*	.49	-.04	.11	.13	.13	.10
	Working relationship	r	.41	-.53	-.41	.20	-.20	-.41	.41	.53	.82*	.35	-.12	.37	.18	.18	.04
	Accountability	r	-.20	-.36	-.61	.41	.20	-.61	-.20	.53	.39	.32	.06	-.09	-.18	-.18	-.26
Female	Total	r	.41	-.49	-.61	.20	.00	-.61	.41	.40	.67*	.45	-.04	.11	.13	.13	.18
	Working relationship	r	.61	-.49	-.61	.20	.00	-.61	.61	.22	.67*	.59	-.18	.11	.13	.13	.35
	Accountability	r	.20	-.49	-.61	.41	.00	-.61	.20	.40	.67*	.49	-.04	.11	.13	.13	.10
Male	Total	r	-.20	-.40	-.41	.41	.00	-.41	-.20	.67	.54	.22	-.02	.17	-.13	-.13	-.39
	Working relationship	r	.00	-.40	-.41	.41	-.20	-.41	.00	.67	.71*	.26	-.02	.30	.04	.04	-.22
	Accountability	r	-.41	-.18	-.61	.41	.20	-.61	-.41	.53	.22	.18	.24	-.22	-.18	-.18	-.30

**Table 5b:** Spearman's rank correlation coefficient (r) between JeffSATIC-J score and length of courses related to teamworking or clinical practice (B) JeffSATIC-J score of sixth-year students

		Institution	Courses related to teamwork															Clinical practice	
			First-fourth year					Fifth year			Sixth year after clinical clerkship	First-sixth year					First-fourth year	Fifth-sixth year	
			UPE	MPE	IPE	M/IPE	Total	UPE	MPE	IPE	IPE	UPE	MPE	IPE	M/IPE	Total	Observation	Clerkship total	Community
			(hrs)	(hrs)	(hrs)	(hrs)	(hrs)	(hrs)	(hrs)	(hrs)	(hrs)	(hrs)	(hrs)	(hrs)	(hrs)	(hrs)	(hrs)	(weeks)	(weeks)
Length of courses		GU	0	4.25	1.75	6	6	0	0	0	0	0	4.25	1.75	6	6	0	62	10‐14
		KyuU	0	9‐22	7‐23	16-45	16-45	3.3	0	0	0	3.3	9‐22	7‐23	16-45	19.3-48.3	0	59	1‐ 5
		KaU	15	0	22.5	22.5	37.5	0	0	0	0	15	0	22.5	22.5	37.5	37	59	1‐12
		SU	0	0	28.5	28.5	28.5	0	0	0	0	0	0	28.5	28.5	28.5	0	50	1‐ 9
		KiU	0	22.5	0	22.5	22.5	0	0.5	10.4	0	0	22.5	10.4	33.4	33.4	106	45	0‐ 6
		KMU	3	0	20	20	23	0	0	0	0	3	0	20	20	23	40	52	2
		JMU	1.2	0	0	0	1.2	0	0	0	5.8	1.2	0	5.8	5.8	7	0	74	7‐15
Overall	Total	r	.20	.02	-.64	-.80*	-.80*	.00	-.20	-.20	.61	.19	.02	-.87**	-.80*	-.76*	-.35	.88**	
	Working relationship	r	.06	-.08	-.21	-.53	-.53	.41	-.41	-.41	.61	.37	-.08	-.39	-.53	-.40	-.67*	.78*	
	Accountability	r	.20	.02	-.64	-.80*	-.80*	.00	-.20	-.20	.61	.19	.02	-.87**	-.80*	-.76*	-.35	.88**	
Female	Total	r	.24	-.22	-.21	-.53	-.53	.20	-.41	-.41	.61	.41	-.22	-.39	-.53	-.40	-.57	.78*	
	Working relationship	r	.18	-.12	.19	-.18	-.18	.61	-.61	-.61	.20	.67	-.12	-.20	-.18	-.13	-.61	.67	
	Accountability	r	.06	.02	-.56	-.80*	-.80*	.00	-.20	-.20	.61	.07	.02	-.77*	-.80*	-.76*	-.49	.85**	
Male	Total	r	.16	-.02	-.51	-.80*	-.80*	.20	-.41	-.41	.61	.26	-.02	-.87**	-.80*	-.76*	-.49	.96***	
	Working relationship	r	-.12	.02	-.28	-.67	-.67	.41	-.41	-.41	.61	.19	.02	-.53	-.67	-.58	-.77*	.83*	
	Accountability	r	.33	-.16	-.51	-.80*	-.80*	.00	-.41	-.41	.61	.30	-.16	-.87**	-.80*	-.76*	-.39	.96***	

JeffSATIC-J: Japanese version of the Jefferson Scale of Attitudes Toward Interprofessional Collaboration; GU: Gifu University School of Medicine; KyuU: Kyushu University School of Medicine; KaU: Kagoshima University School of Medicine; SU: Showa University School of Medicine; KiU: Kitasato University School of Medicine; KMU: Kanazawa Medical University School of Medicine; JMU: Jichi Medical University School of Medicine; UPE: education for medical students only (uniprofessional), in which medical students learn without any other health professions students; MPE: multiprofessional education without mutual interaction between students of different professions; IPE: multiprofessional education with mutual interaction between students of different professions, which students learn from and about each other; M/IPE: MPE and IPE. r: Spearman's rank correlation coefficient. *p < .05, ** p < .01, *** p< .001

## Discussion

We developed the JeffSATIC-J, which is equivalent to the original JeffSATIC. Surveys with the JeffSATIC-J revealed that female students and first-year students had more positive attitudes toward collaborative teamwork than male students and fourth- and sixth-year students who learned about teamwork as part of their formal curriculum. Based on a theoretical and empirical study review, San Martín-Rodríguez and colleagues elucidated three influential factors for successful team collaboration: systemic, organizational, and interactional determinants.^30^ Using these determinants, the construction of medical trainees' collaborative attitudes targeted in this research is discussed below.

The JeffSATIC-J score of Japanese medical students in this multiple imputation study was 107.4 (SD 13.9), whereas previously reported JeffSATIC scores of US medical students were 115.5 (SD 12.3, n = 219) at Jefferson University and 115.5 (SD 18.7, n = 115) at Midwestern University.^25^ As the effect size was 0.58, a significant difference between this and previous US studies was suggested. Onishi and colleagues, based on JSAPNC scores, reported that Japanese doctors had lower recognition of collaboration than US doctors.^31^ This may suggest that Japanese students as well as doctors have more negative attitudes toward collaboration than US students.

Hojat and colleagues concluded that the differences between the countries might be due to cultural reasons.^27^ Furthermore, the professional system, another systemic determinant for team collaboration, must be considered. The Japanese Medical Practitioners' Act states, "No person except a medical practitioner shall engage in medical practice" (Article 17)^32^ and medical practice is strictly limited to nationally licensed medical doctors who graduate from medical school (Article 11) and pass the National Examination for Medical Practitioners administered by the Minister of Health and Labour (Article 9). However, non-physician clinicians in the US, such as physician assistants and nurse practitioners, can perform medical acts on behalf of physicians. Japanese culture and a professional system that formally establishes the professional superiority of licensed medical doctors can help explain the negative attitudes toward collaboration among Japanese medical students and doctors.

In contrast, female students' high collaborative attitudes were indicated in this research and repeatedly reported from the US and Sweden.^25^^,^^29^ However, gender was not a significant factor for residents' "working relationship" scores in this study, suggesting that environment, education, and clinical experiences could help modify collaborative attitudes. In addition to regional culture, organizational determinants, such as each organization's philosophy and shared values, and experiences provided by the formal educational program and informal activities at the institution might cause differences in attitudes toward collaboration. Organizational determinants could also explain differences in medical students' attitudes among institutions and learning year groups.

Hansson and colleagues reported that collaborative attitudes in Swedish medical students, as evaluated by the JSAPNC, were significantly more negative in the final year of medical school than in the first year.^29^ In our study, first-year students' collaborative attitudes were higher than those of fourth- and sixth-year students, and sixth-year students' attitudes were close to those of residents and doctors. The results of the current as well as previous studies suggest that students gradually acquire an attitude as medical doctors, including attitudes toward team collaboration in the medical community.^29^ This may be due to students' ambitions to identify with the doctor's role and its demarcation from other professional groups.^29^^,^^33^^,^^34^ Educational intervention might modify students' attitudes. IPE is known to be an effective educational strategy to enhance awareness of team collaboration as indicated by educational outcomes and the adoption of collaborative experiences.^9^^-^^11^

In this study, IPE had a positive but short-term effect on female students' collaborative attitude, and clinical experience had a positive effect on both male and female students, especially on their "accountability" with respect to collaborative attitudes at graduation. The clinical experience provided students with the opportunity to realize the necessity of performing team collaboration and to gain respect for other health professionals.

Harden^13^ explained that learning in clinical clerkship courses is a transprofessional step of teamwork education, which is more advanced than multiprofessional or interprofessional steps. Clinical clerkship courses in Japanese medical schools correspond to Harden's transprofessional step of education. Frenk and colleagues indicated the importance of transprofessional education in communities with multiple health workers.^35^ These learning experiences were categorized as interactional determinants for successful team collaboration,^30^ and this research supports the effectiveness of transprofessional experiences.

JMU has a unique clinical clerkship following the IPE course, and sixth-year students at JMU had the highest JeffSATIC-J scores among the seven medical schools in this study. Wahlström and colleagues reported that long-term education provided in the context of the clinical practice of medicine improves attitudes and skills related to collaboration with other health professions.^36^ Previous research indicates that informal interprofessional interactions during clinical placements serve as an effective method of team learning.^37^^,^^38^ Frenk and colleagues suggested that interprofessional undergraduate education should be integrated into socialization and learning as part of a continuum of learning.^35^ JMU students' positive attitude toward collaboration might have been constructed by modeling and experiencing favorable teamwork as part of their clinical clerkship courses, and then reinforced by experienced-based learning in interprofessional group discussions. Students' rich clinical experience as health professionals, interacting with people who would be team members in the near future, combined with reflective learning in the formal course program might foster ideal team collaborators.

### Limitations

Despite conducting self-assessment using the JeffSATIC-J in an anonymous survey, the possibility of social desirability bias cannot be ruled out in this study. Courses were categorized based on the CAIPE definition and Harden's educational steps and compared by the length of the courses. Although the length of the courses could be a significant factor, courses varied among the medical schools studied despite their classification into the same category, and confounding factors might influence scale scores.

Further, the formal course curriculum was used to determine 6 years of educational intervention. Course information might not reflect actual individual educational experiences, and the method of statistical analysis was limited because there was an elective course at KyuU. As for residents and physicians, the number of institutions was limited, and respondents' undergraduate educational backgrounds were unknown. As this was a cross-sectional study, our conclusions should be discussed carefully and confirmed in a cohort study.

## Conclusions

Japanese medical trainees' collaborative attitude toward teamwork might be lower than that of US students, and could be influenced by culture, professional systems, and organizational factors. Although students in their final year of medical school have lower collaborative attitudes toward teamwork than first-year students, experience in clinical clerkships might facilitate their recognition as well as the execution of collaborative work. Transprofessional education in the community of practice and an effectively organized curriculum might have favorable effects on medical students' attitudes toward collaboration.

The results of this study suggest that long-term clinical clerkship at higher grades need to be implemented in the curriculum to improve final-year medical students' attitudes toward team collaboration. Further work is required to reveal details of the related factors such as focus group interviews.

### Acknowledgments

We are grateful to the respondents and everyone who supported this research.

### Conflict of Interest

The authors declare that they have no conflict of interest.
